# Aging and mental health of the Chornobyl catastrophe survivors

**DOI:** 10.1192/j.eurpsy.2022.1518

**Published:** 2022-09-01

**Authors:** N. Gunko, K. Loganovsky, V. Buzunov, N. Korotkova

**Affiliations:** 1 State Institution “National Research Centre for Radiation Medicine, National Academy of Medical Sciences of Ukraine”, Laboratory Of Medical Demography, Department Of Epidemiology, Institute Of Radiation Hygiene And Epidemiology, Kyiv, Ukraine; 2 State Institution “National Research Centre for Radiation Medicine of National Academy of Medical Sciences of Ukraine”, Radiation Psychoneurology, Institute Of Clinical Radiology, Kyiv, Ukraine; 3 State Institution “National Research Centre for Radiation Medicine, National Academy of Medical Sciences of Ukraine”, Epidemiology, Institute Of Radiation Hygiene And Epidemiology, Kyiv, Ukraine

**Keywords:** registers, mental health, Chornobyl catastrophe survivors, demographic aging

## Abstract

**Introduction:**

The Chornobyl catastrophe (ChC) has impacted on depopulation and mental health of the survivors, however, the rate of demographic aging and mental health survey remain at issue.

**Objectives:**

To determine the rate of demographic aging of ChC survivors and to analyze the state of their mental health survey.

**Methods:**

Information from the Ministry of Health and the State Statistics Service of Ukraine, Clinical and Epidemiological Register, the State Register of ChC survivors, and clinical neuropsychiatric data were analyzed.

**Results:**

In 2018, compared to 1995, the number of the ChC survivors decreased by 987 thousand with high level of aging: ChC clean-up workers – 59.0%; evacuees – 25.0%, and residents of radioactively contaminated territories (RCT) – 30.7%. There are negative tendencies in age parameters of survival of the RCT population. Long-term mental health disorders and neuropsychiatric effects in the ChC survivors have been identified, which may indicate an accelerated aging. Neurophysiological and molecular-biological atypia of aging processes under an exposure to low doses of and low dose rate of ionizing radiation have been found. Existing statistical and registry data underestimate the level of mental disorders in the population of Ukraine, including the ChC survivors by an order of magnitude.

**Conclusions:**

The negative tendencies in age parameters of survival indicate the need to continue research to identify the factors “responsible” for such changes. Mental health disorders and neuropsychiatric effects in the ChC survivors are underestimated. It is necessary to create a national psychiatric registry of Ukraine and long-term (lifelong) monitoring of survivors.

**Disclosure:**

No significant relationships.

